# DNA methylation meningioma biomarkers: attributes and limitations

**DOI:** 10.3389/fnmol.2023.1182759

**Published:** 2023-07-10

**Authors:** Zhaohui Li, Yufei Gao, Jinnan Zhang, Liang Han, Hang Zhao

**Affiliations:** ^1^Department of Neurosurgery, China-Japan Union Hospital of Jilin University, Changchun, China; ^2^Department of Pathology, China-Japan Union Hospital of Jilin University, Changchun, China

**Keywords:** DNA methylation inhibitors, genomics, methylated biomarker, meningioma, WHO grades

## Abstract

Meningioma, one of the most common primary central nervous system tumors, are classified into three grades by the World Health Organization (WHO) based on histopathology. The gold-standard treatment, surgical resection, is hampered by issues such as incomplete resection in some cases and a high recurrence rate. Alongside genetic alterations, DNA methylation, plays a crucial role in progression of meningiomas in the occurrence and development of meningiomas. The epigenetic landscape of meningioma is instrumental in refining tumor classification, identifying robust molecular markers, determining prognosis, guiding treatment selection, and innovating new therapeutic strategies. Existing classifications lack comprehensive accuracy, and effective therapies are limited. Methylated DNA markers, exhibiting differential characteristics across varying meningioma grades, serve as invaluable diagnostic tools. Particularly, combinatorial methylated markers offer insights into meningioma pathogenesis, tissue origin, subtype classification, and clinical outcomes. This review integrates current research to highlight some of the most promising DNA and promoter methylation markers employed in meningioma diagnostics. Despite their promise, the development and application of DNA methylation biomarkers for meningioma diagnosis and treatment are still in their infancy, with only a handful of DNA methylation inhibitors currently clinically employed for meningioma treatment. Future studies are essential to validate these markers and ascertain their clinical utility. Combinatorial methylated DNA markers for meningiomas have broad implications for understanding tumor development and progression, signaling a paradigm shift in therapeutic strategies for meningiomas.

## Introduction

Meningioma is a type of brain tumor that arises from the meninges, the protective membranes that surround the brain and spinal cord, and one of the most common primary central nervous system tumors. World Health Organization (WHO) classifies it into three grades based on histopathology (Pereira et al., 2019). Global incidence of operated meningiomas has 4.51 for 100000 person and WHO grade I meningiomas occur for two-thirds in females (Champeaux et al., 2019). Clinical therapy of meningiomas includes surgery, radiotherapy, chemotherapy, and/or their combination. However, some meningiomas remain challenging to be cured and inclined to recurrence. Surgical resection is the preferred treatment for meningiomas, but there are problems such as inability to obtain total resection in some patients and high postoperative recurrence rate of high-grade meningiomas. The histological categorization of meningiomas by the World Health Organization (WHO) consists of Grade I Benign, Grade II Atypical, and Grade III Anaplastic. Nonetheless, meningiomas are largely histomorphology and varieties are unlimited, such as the degree of mitotic activity, brain invasion, and aggressive histologic activities. Current WHO classifications of meningiomas are limited to predict benign tumors and aggressive ones, and fail to precisely foretell the clinical performance, aggressiveness, and long-period recurrence of specific meningiomas (Hergalant et al., 2022).

Recent meningioma biomarker analyses make it possible to make more recognizable meningioma subtypes, their performance, and the prospective for innovative therapies (Cao et al., 2023). Preceding study engaging in targeted sequencing or histochemical examination has helped to illuminate the genetic patterns of meningiomas. The identification of particular genetic modifications allows for additional sorting of meningiomas into more clear subgroups, thus providing more improved diagnosis, prognosis, and possible therapy. In addition to genetic changes such as gene mutations and chromosomal deletions, epigenetics also play an important role in the occurrence and development of meningiomas (Daoud et al., 2022). The epigenetic characteristics of meningioma is of great significance for the classification of meningioma, the discovery of reliable molecular markers, the judgment of prognosis, the selection of treatment options, and the discovery of new treating methods. DNA methylation is a common epigenetic modification that involves the addition of a methyl group to cytosine residues in DNA, which can regulate gene expression and contribute to the development of cancer. Recent research has identified several DNA methylation biomarkers that are associated with meningioma. These meningiomas harboring DNA methylation subgroups have distinctive biological and clinical features (Choudhury et al., 2022).

It’s worth noting that the use of DNA methylation biomarkers for meningioma diagnosis and treatment is still in the early stages. Further research is needed to validate these biomarkers and to determine their clinical utility. The goal of the present review is to discuss attributing potential of methylated meningioma biomarkers, the space for the potential clinical application, and future efforts should be focused.

## DNA methylation biomarkers of meningiomas

DNA methylation can affect gene expression and cellular function. Aberrant DNA methylation has been linked to the development and progression of many types of subtypes in meningiomas. The WHO classification (2016) of meningiomas is divided into three grades (Louis et al., 2016): Grade I, this includes meningothelial, fibrous (fibroblastic), transitional (mixed), psammomatous, angiomatous, microcystic, secretory, lymphoplasmacyte-rich, and metaplastic meningiomas; Grade II (atypical), this includes chordoid, clear cell, and atypical meningiomas and Grade III (anaplastic/malignant), this includes papillary, rhabdoid, and anaplastic meningiomas. Each grade is associated with a different risk of recurrence, with Grade I tumors having the lowest risk and Grade III tumors having the highest risk. In this system, grade II and III tumors are characterized by increased mitotic activity or the presence of specific malignant histological features. Atypical meningiomas (grade II) show either increased mitotic activity or at least three of the following criteria: increased cellularity, small cells with a high nuclear: cytoplasmic ratio, prominent nucleoli, uninterrupted patternless or sheet-like growth, or foci of necrosis. Anaplastic meningiomas (grade III) are histologically malignant tumors with high mitotic activity.

The article by Louis et al. provides a comprehensive overview of the fifth edition of the WHO Classification of Tumors of the Central Nervous System (WHO CNS5) (Louis et al., 2021). This classification is the international standard for the classification of brain and spinal cord tumors. The WHO CNS5 builds on the updated fourth edition that appeared in 2016, incorporating numerous molecular changes with clinicopathologic utility that are important for the most accurate classification of CNS neoplasms. Meningioma is considered a single type in WHO CNS5, with its broad morphological spectrum reflected in 15 subtypes. It is now emphasized that the criteria defining atypical or anaplastic (i.e., grade 2 and 3) meningioma should be applied regardless of the underlying subtype.

The recent article discusses that the most meningiomas are WHO grade 1 and benign. Meningiomas are found in adults with a female predominance (male-to-female ratio, 1:3). Sellar or parasellar meningiomas often occur in the tuberculum, diaphragma, and dorsum sellae, and planum sphenoidale, clinoid processes, and cavernous sinuses. Purely intrasellar meningiomas are rare, develop from the periphery, and extend into the sella turcica. In terms of imaging findings, meningiomas usually show isointensity to gray matter on T1WI, slight hyperintensity to hypointensity to gray matter on T2WI, and strong and uniform contrast enhancement, often with a dural tail. CT may show calcification within the tumor, bony hyperostosis, or an enlarged sphenoid sinus (pneumosinus dilatans) in adjacent regions. The factors distinguishing it from PitNET/pituitary adenoma are that it is more common in women, presents with a normal (compressed) pituitary gland, rarely shows a snowman shape, and has no sellar dilation, and uniform contrast enhancement is observed, a dural tail is frequently observed, bony hyperostosis is present in adjacent regions, and the ADC is higher than that of PitNET/pituitary adenoma (Tsukamoto and Miki, 2023).

DNA methylation biomarkers can provide valuable information about the diagnosis, prognosis, and response to treatment of meningiomas. One important reason for exploring DNA methylation biomarkers in meningiomas is to improve their classification and grading. Meningiomas are currently classified based on their histology and graded based on their aggressiveness, which can guide treatment decisions. However, the accuracy of histological grading is limited, and there is considerable variability. DNA methylation biomarkers can provide more objective and reliable information about the biological behavior of meningiomas and help identify those that are more likely to progress or recur. In addition, DNA methylation biomarkers can provide valuable information about the response to treatment. Meningiomas are typically treated with surgery, radiation therapy, and/or chemotherapy. However, there is considerable variability in treatment response, and some tumors may not respond to therapy or recur after treatment. DNA methylation biomarkers can help identify those patients who are more likely to respond to a particular treatment and help guide treatment decisions.

Atypical (WHO grade II) and malignant meningiomas (WHO grade III) have a high recurrence rate after surgical resection and radiotherapy. This has led to an interest in exploring other systemic treatment options for these refractory tumors. Several clinical trials are currently recruiting patients to translate targeted molecular therapy for recurrent and high-grade meningiomas. High-grade meningiomas, particularly anaplastic tumors, show a reduction in the count of CD4+, CD8+, and PD-1+ T cells, and an increase in the number of FoxP3+ T-regulatory cells (Tregs). This immune cell phenotype is associated with tumor-mediated evasion of the immune system. The classical first-line treatment for all meningiomas is surgery. However, high-grade meningiomas have a high recurrence rate; up to 60% of tumors may recur after 15 years of complete resection. The use of systemic treatments as standard care remains experimental and is reserved for cases of recurrent/progressive disease not suitable for surgery or radiotherapy. In conclusion, the article supports the statement that some meningiomas may not respond to therapy or recur after treatment, specifically WHO grade II (atypical) and Grade III (anaplastic) meningiomas, and often exhibit therapy resistance and higher recurrence rates (Garcia-Robledo et al., 2021).

### Single-gene DNA methylation of meningioma biomarkers

Neurofibromatosis type 2 (NF2) is an inherited genetic disorder that increases the risk of developing tumors of the nervous system. The gene responsible for *NF2* is located on chromosome 22, and mutations in this gene can lead to the development of meningiomas. DNA methylation of the *NF2* gene, as well as other genes involved in cell cycle regulation and tumor suppression, may be altered in meningioma patients (Table 1; Bujko et al., 2017). Suppressor of fused homolog (SUFU), a strong inhibitor of the transcription factors GLI (Drosophila’s Krüppel-like zinc finger protein). SUFU showed values lower than normal in meningioma (Laurendeau et al., 2010). DNA methylation of *SUFU* has been studied in meningioma. Hypermethylation of *SUFU* was associated with a higher tumor grade and patients (Laurendeau et al., 2010).

**Table 1:** DNA methylation of meningioma biomarkers.

Meningioma marker(s)	WHO classification	Methylation classification	Function	References
Single gene DNA methylation				
*NF2*	Grade I, II and III	All methylation	Fibrous type meningioma; tumor sizes with *NF2* marker are larger than tumors with other markers	Yuzawa et al. (2016)
*POL2RA*	Grade I	NA	High likelihood of meningioma recurrence	Clark et al. (2016)
*SUFU*	Grade II and III	Int-B, Mal	SUFU showed values lower than normal in meningioma.	Laurendeau et al. (2010)
*POLR2A*	Grade I	All methylation	POLR2A alterations was uncovered exclusively in benign tumors	Preusser et al. (2018)
*FAK*	Grade II and III	All methylation	Focal adhesion kinase (FAK) inhibition has been shown to exert antitumor activity in Phase II of meningioma.	Brastianos et al. (2023)
*WINK2*	Grade I, II and III		Aberrant methylation of the CpG island of WINK2 was associated with decreased expression in primary tumors	Shen et al. (2020)
*MAL2*	Grade I, II and III	All methylation	*MAL2* promoter region leads to the downregulation of MAL2 expression in meningioma cells, which is associated with a more aggressive tumor phenotype and a worse prognosis for patients.	Canisius et al. (2022)
*MLLT10*	Grade I, II and III	All methylation	MLLT10 promoter methylation was found in 36.8% of the meningioma samples, and this methylation was significantly associated with higher tumor grade and a higher rate of recurrence.	Walsh et al. (2022)
*RIC8A*	Grade I, II and III	All methylation	RIC8A was significantly hypermethylated in the tumor samples compared to normal brain tissue.	Liu et al. (2020)
*BRAF mutation (V600E)*		All methylation	BRAF V600E mutant rhabdoid meningiomas have been reported to present an aggressive clinical course	El-Habr et al. (2014)
Multiple gene DNA methylation				
*NF2, ARID1A, SRSF2*	Grade III (rhabdoid meningioma)	All methylation	Meningioma development and progression	Bujko et al. (2017)
*NF2, SMARCB1*	Grade I, II and III	All methylation	Both NF2 and SMARCB1 was associated with a higher risk of meningioma recurrence than either gene alone, to distinguish between different subtypes of meningioma, and had a poorer prognosis than those with only one or neither gene methylated.	Smith (2015)
*TRAF7, KLF4, AKT1*	Grade I	Ben-2, Ben-3, Int-B	Mutations involved in PI3K signaling, secretory type meningioma	Clark et al. (2013)
*PIK3CA, TRAF7*	Grade I	Ben-2, Ben-3, Int-B	Mutations involved in PI3K signaling	Smith (2015)
*SMO, SUFU, PRKAR1A*	Grade I	Ben-2, Int-B, Mal	Mutations involved in sonic hedgehog signaling (Shh)	Clark et al. (2013)
*TRAF7， KLF4*	Grade I	Ben-2	Combined TRAF7/KLF4 mutations invariably predict the secretory subtype.	Abedalthagafi et al. (2016)
*AKT1， SMO*	Grade I	Ben-2	AKT1 and SMO mutations constitutively activate cellular proliferation pathways.	Abedalthagafi et al. (2016)
*AKT1, PI3KCA*	Grade I	Ben-3	AKT1 mutation upregulates PI3K/AKT/mTOR signaling pathway while PI3K is affected in mutations involving PI3KCA constitutively activating the pathway	Goyal-Honavar et al. (2022)
*SMARCB1, SMARCE1*	Grade II and III	All methylation	SMARCB1 and SMARCE1 have all been associated with a predisposition to meningiomas	John et al. (2022)
*CDKN2A and CDKN2B*	Grade III	Mal	*CDKN2A* and CDKN2B regulate cell apoptosis via p53 pathway.	Smith (2015)
*DMD*	Grade III	All methylation	A more aggressive form of meningioma.	Lee and Lee (2020)
*NF2, p14 (ARF), CDH1, BRCA1, RB1*		All methylation	Methylation was detected in *NF2*, p14ARF, CDH1, BRCA1, RB1 previously shown to be silenced in meningioma.	Banerjee et al. (2002)
*p73, RASSF1A*	Grade I, II and III	All methylation	Tumor size, invasion, grade and recurrence of meningiomas	Paramasivam et al. (2019)
*HOXA 7, 9, and 10*	Grade I, II and III	All methylation	The methylation of HOXA 7, 9, and 10 were associated with Histopathology and aggressiveness parameters	van Tilborg et al. (2006)
*IGF2BP1 and PDCD1*	Grade I, II and III	All methylation	Malignant potential, PFS and OS	He et al. (2020)
*NF2, MN1, ARID1B, SEMA4D, and MUC2*	Grade III	All methylation	Development and subtype classification of meningiomas	Murnyák et al. (2015) and Gao et al. (2013)
*p53, p14ARF, MEG3*		All methylation	Meningioma progression	Vengoechea et al. (2013)
*CDKN2A, NDRG2,TIMP3*		All methylation	Meningioma progression and recurrence.	Liu et al. (2020)
*MGMT, WNK2*		All methylation	Meningioma development and progression.	Zhang et al. (2010)

DNA methylation is stratified to six biological (methylation classes benign ben-1, 2, and 3, methylation class intermediate int-A and B, and methylation class malignant mal) and three combined clinical MCs (benign, intermediate, malignant) (Sahm et al., 2017). AKT1, v-Akt murine thymoma viral oncogene homolog 1; ARID1A, AT-rich interactive domain-containing protein 1A; ARID1B, AT-rich interactive domain-containing protein 1B; BRAF mutation (V600E), v-raf murine sarcoma viral oncogene homolog B1; BRCA1, Breast cancer type 1 susceptibility protein; CDH1, Cadherin-1; CDKN2A, Cyclin Dependent Kinase Inhibitor 2A; CDKN2B, Cyclin Dependent Kinase Inhibitor 2B; CHEK2, Checkpoint kinase 2; DMD, Dystrophin; FAK, Focal adhesion kinase; HOXA, homeobox A; IGF2BP1, Insulin-like growth factor 2 mRNA-binding protein 1; KLF4, Kruppel-like factor 4; MAL2, myelin and lymphocyte protein 2; MEG3, Maternally expressed gene 3; MGMT, O[6]-methylguanine-DNA methyltransferase; MLLT10, myeloid/lymphoid or mixed-lineage leukemia; MN1, meningioma 1; MUC2, Mucin 2; NDRG2, N-Myc Downstream-Regulated Gene 2; NF-2, neurofibromatosis 2; OS, overall survival; p14 (ARF), Cyclin-dependent kinase inhibitor 2A; PDCD1, Programmed Cell Death 1; PFS, progression-free survival; PI3KCA, Phosphatidylinositol 3-kinase; POLR2A, DNA directed RNA polymerase II A; RASSF1A, Ras association domain family protein 1 isoform A; RB1, Retinoblastoma protein; SEMA4D, Semaphorin 4D; SMO, Smoothened, frizzled class G protein-coupled receptor; SMARCB1, SWI/SNF-Related, matrix-associated, actin-dependent regulator of chromatin subfamily B member 1; SMARCE1, SWI/SNF Related, matrix-associated, actin-dependent regulator of chromatin subfamily E member 1; SRSF2, Serine/arginine-rich splicing factor 2; SUFU, suppressor of fused; THBS1, Thrombospondin-1; TIMP3, Tissue inhibitors of metalloproteinase 3; TRAF7, Tumor necrosis factor receptor-associated factor 7; WNK2, WNK lysine deficient protein kinase 2.

*POLR2A*, also known as RNA Polymerase II subunit A is a gene that encodes the largest subunit of RNA Polymerase II, which is responsible for transcribing mRNA. *POLR2A* alterations was uncovered exclusively in benign tumors (Preusser et al., 2018). Hypermethylation of *POLR2A* was associated with a higher risk of meningioma, suggesting that DNA methylation of POLR2A may play a role in the development of meningioma (Clark et al., 2016). Focal adhesion kinase (*FAK*) inhibition has a synthetic lethal relationship with *NF2* loss. Considering the predominance of *NF2* mutations in meningiomas, GSK2256098 (a FAK inhibitor) was used in phase II study in recurrent or progressive grade 1–3 meningiomas (Brastianos et al., 2023). *FAK* inhibition has been shown to exert antitumoral activity in *in vitro* meningioma models (Mair et al., 2022).

*DMD* (Dystrophin) is a gene located on the X chromosome that encodes for the dystrophin protein, which plays a critical role in muscle function. Mutations or changes in the methylation status of DMD have been associated with several disorders, including Duchenne muscular dystrophy and Becker muscular dystrophy. DMD was one of several genes that were hypermethylated in the tumors. The researchers also found that DMD methylation was associated with a more aggressive form of meningioma (Paramasivam et al., 2019). DMD can be a biomarker for meningiomas, specifically for progressive/high-grade meningiomas. DMD inactivation was found in 32% of progressive meningioma patients in one study. Importantly, patients with tumors harboring DMD inactivation had a shorter overall survival than those without this alteration. In a multivariate model, DMD inactivation and TERT alterations were mutually independent in predicting unfavorable outcomes (Juratli et al., 2018).

WNK lysine deficient protein kinase 2 (*WINK2*) is involved in the regulation of ion transport, particularly of sodium, potassium, and chloride ions, in various tissues including the kidney and the nervous system. Aberrant methylation of the CpG island of *WINK2* was associated with decreased expression in primary tumors (Shen et al., 2020). The hypermethylation of WNK2 was found in 83% of grade II and 71% of grade III meningiomas, and was associated with loss of gene expression. Since WNK2 acts as a negative regulator of cell proliferation, loss of WNK2 is likely associated with more aggressive tumor growth (Jun et al., 2009).

Myelin and lymphocyte protein 2 (MAL2) have been implicated in a variety of cellular processes, including the regulation of membrane trafficking, cell signaling, and cell adhesion. In the nervous system, MAL2 has been shown to play a role in the formation and maintenance of myelin, the fatty substance that surrounds and insulates nerve fibers and allows for efficient transmission of electrical signals between neurons. *Mal2* DNA methylation was found to be associated with proliferation, migration, and invasion in meningioma (Canisius et al., 2022). Myeloid/lymphoid or mixed-lineage leukemia (MLLT10) is known to be involved in several biological processes, including transcriptional regulation, DNA repair, and cell cycle control. *MLLT10* DNA methylation is positively associated with the risk of female meningioma (Walsh et al., 2022). *RIC8A* is a protein-coding gene that plays a role in the regulation of G protein signaling, which is involved in various physiological processes such as vision, smell, and the response to hormones and neurotransmitters. The *RIC8A* DNA methylation is associated with pathological phenotypes in meningioma (Liu et al., 2020).

*BRAF* is a gene that encodes a protein called B-Raf, which is a part of a cellular signaling pathway known as the MAP kinase pathway. Mutations in *BRAF* have been associated with a number of meningioma. In meningioma, a specific mutation in *BRAF* gene, known as the V600E mutation, has been found in a subset of meaningioma. This mutation results in a change in the amino acid sequence of the B-Raf protein, which leads to its increased activity and constitutive activation of the MAP kinase pathway. This can contribute to the development and progression of the meningioma. *BRAF* V600E mutant rhabdoid meningiomas have been reported to present an aggressive clinical course, was improved with BRAF inhibitor (dabrafenib) (Lee and Lee, 2020).

### Multiple-gene DNA methylation of meningioma biomarkers

AT-rich interactive domain-containing protein 1A (*ARID1A*) is a gene that encodes a protein involved in chromatin remodeling. DNA methylation alterations of *ARID1A* may be involved in the development and progression of meningiomas. *ARID1A* is frequently mutated or deleted in meningiomas. The level of *ARID1A* expression is reduced in these tumors, which is associated with poorer clinical outcomes. Serine/arginine-rich splicing factor 2 (*SRSF2*) is a splicing factor that has also been implicated in cancer development. Increased methylation of the *SRSF2* gene has been associated with a higher risk of meningioma recurrence and a more aggressive tumor phenotype. DNA methylation of *NF2*, *ARID1A*, and *SRSF2* may play a role in meningioma development and progression (Table 1; Bujko et al., 2017). In a study, it was found that mutations in the ARID1A gene were associated with recurrent meningiomas, indicating that the presence of these mutations in primary tumors could be a predictor of recurrence. Specifically, when ARID1A mutations were present in primary atypical meningiomas (WHO grade II), the hazard of recurrence increased by 625% (HR = 7.26 [1.42–37.0]; *p* = 0.017). The study concluded that these tumors tended to have worse prognosis, suggesting that ARID1A could potentially be a prognostic biomarker for meningioma. However, the research also highlighted that further prospective studies would be needed to validate ARID1A as a prognostic marker. This indicates that while the initial results are promising, more research is required to confirm ARID1A’s utility as a biomarker (Chaluts, 2022).

*NF2* and SWI/SNF-Related, matrix-associated, actin-dependent regulator of chromatin subfamily B member 1 (*SMARCB1*) are tumor suppressor genes that have been implicated in the development of meningiomas. *NF2* is frequently mutated or lost in meningiomas, and *SMARCB1* has been shown to be frequently inactivated in some meningiomas, particularly those that are classified as rhabdoid meningiomas. The methylation of both *NF2* and *SMARCB1* was associated with a higher risk of meningioma recurrence than either gene alone. The combination of *NF2* and *SMARCB1* methylation status could be used to distinguish between different subtypes of meningioma, and that patients with both genes methylated had a poorer prognosis than those with only one or neither gene methylated. DNA methylation of both *NF2* and *SMARCB1* may play an important role in meningioma development and progression, and that their combined methylation status could be a useful biomarker for meningioma diagnosis and prognosis (Table 1; Smith, 2015).

TNF receptor-associated factor 7 (TRAF7) produces a proapoptotic E3 ubiquitin ligased in meningiomas, which is critically associated with activation of the nuclear factor-κB (NF-κB), mitogen-activated protein kinase (MAPK), and interferon signaling. It is always linked with Kruppel-like factor 4 (KLF4), V-akt murine thymoma viral oncogene homolog 1 (AKT1), and the phosphatidylinositol 3-kinase (PI3K) mutations. The low-grade, meningothelial subtypes, combined TRAF7/KLF4 mutations, consistently predict the secretory subtype (Table 1; Goyal-Honavar et al., 2022). AKT1 and smoothened, frizzled family receptor (SMO) mutations invariably stimulate cellular proliferation of meaningioma (Champeaux et al., 2019). The AKT1 mutation upregulates PI3K/AKT/mTOR signaling pathway (Yuzawa et al., 2016).

*SUFU*, *SMARCB1* and *SMARCE1* have all been associated with a predisposition to meningiomas and are all known to bind the Shh pathway transcription factor, Gli1 (Smith, 2015; Lee and Lee, 2020). *SMARCB1* and *SMARCE1* are two genes that are important for the regulation of gene expression and chromatin remodeling. Mutations or abnormal DNA methylation in these genes have been associated with the development of meningioma. In meningiomas, hypermethylation of *SMARCB1* and *SMARCE1* has been associated with a loss of these genes, which can disrupt the normal function of the SWI/SNF complex, which can lead to the aberrant expression of other genes (Smith, 2015).

The cyclin dependent kinase inhibitor 2A (*CDKN2A*) and cyclin dependent kinase inhibitor 2B (*CDKN2B*) genes are located on chromosome 9p21 and code for proteins that act as suppressors by regulating the cell cycle. CDKN2A and CDKN2B, as tumor suppressors, regulate cell apoptosis through modulation of the p53 pathway in meningioma (Banerjee et al., 2002). *CDKN2A* and *CDKN2B* were frequently methylated in meningiomas, and the methylation was associated with a higher grade and recurrence of the tumors. DNA hypermethylation of *CDKN2A* and *CDKN2B* was more common in atypical and aplastic meningiomas compared to benign meningiomas (Banerjee et al., 2002).

The genes *NF2, p14* (*ARF* or *CDKN2A*), cadherin-1 (*CDH1*), breast cancer type 1 susceptibility protein (*BRCA1*), and retinoblastoma protein (*RB1*) are all tumor suppressor genes that have been implicated in meningiomas, which have shown to be frequently altered or mutated. The silence of *NF2, p14 (ARF)*, *CDH1*, *BRCA1*, and *RB1* has been shown to play a role in the development and progression of meningiomas. Methylation of these genes holds potential as biomarkers for diagnosis and prognosis, pending further research and validation, and potentially targeted therapy of meningiomas (Table 1; van Tilborg et al., 2006). p73 is a tumor suppressor gene that is involved in cell cycle regulation, apoptosis, and DNA repair. Ras association domain family protein 1 isoform A (*RASSF1A*) is another tumor suppressor gene that is involved in regulating cell growth Studies have suggested that the combination of p73 and RASSF1A methylation may be a useful biomarker for meningioma diagnosis and prognosis. The methylation status of p73 and *RASSF1A* was significantly associated with tumor size, invasion, grade and recurrence of meningiomas. Specifically, the patients with higher levels of p73 and *RASSF1A* methylation had a poorer prognosis and higher rates of meningioma recurrence (He et al., 2020).

*HOXA* genes are a family of homeobox genes that play important roles in embryonic development and cell differentiation. Abnormal DNA methylation patterns in HOXA genes are associated with meningiomas. DNA methylation levels of *HOXA7*, *HOXA9*, and *HOXA10* were significantly higher in meningioma samples compared to normal meningeal tissue samples. Higher methylation levels of these genes were with a higher grade of meningioma, suggesting that DNA methylation changes in *HOXA* genes may be involved in the development and progression of meningiomas (Table 1; Gao et al., 2013; Murnyák et al., 2015).

Insulin-like growth factor 2 mRNA-binding protein 1 (*IGF2BP1*) is a gene that encodes an RNA-binding protein involved in the regulation of cell growth while programmed cell death 1 (*PDCD1*) encodes a protein called programmed cell death protein 1, which is an immune checkpoint protein that regulates immune responses. Aberrant DNA methylation of these genes has been associated with meningioma. DNA methylation of *IGF2BP1* was significantly higher in meningioma tumors compared to normal tissue, while the methylation of *PDCD1* was lower. DNA methylation levels of *IGF2BP1* were associated with the grade of the meningioma tumor, with higher levels of methylation in higher-grade tumors. Higher levels of *IGF2BP1* methylation were associated with worse clinical outcomes, including shorter PFS and OS (Vengoechea et al., 2013). Methylation of PDCD1 and IGF2BP1 was found to correlate with increased malignant potential and associated with an aggressive phenotype of meningioma (Vengoechea et al., 2013; Garzon-Muvdi et al., 2017).

Mucin 2 (*MUC2*) encodes a protein involved in mucin production and has also been found aberrantly expressed in meningioma. DNA methylation of *NF2, MN1, ARID1B,* Semaphorin 4D (*SEMA4D*)*, and MUC2* have been implicated in the development of meningiomas. The combined analysis of DNA methylation markers has shown promise in classifying meningiomas into different subtypes and identifying unique molecular and clinical characteristics of meningioma (Liu et al., 2020). AT-rich interactive domain-containing protein 1B (ARID1B) mutations found in the cranial (Huntoon et al., 2022). p53 pathways are connected via p14ARF, which is a positive p53 regulator that inhibits MDM2-p53 interaction /tumor grade of meningioma associated with the level of MEG3 (Domingues et al., 2015).

Methylation of the *CDKN2A* gene can lead to reduced expression of these proteins and increased risk of meningioma development. N-Myc downstream-regulated gene 2 (*NDRG2*) is another tumor suppressor gene that regulates various cellular processes, including cell proliferation, differentiation, and apoptosis. Methylation of the *NDRG2* gene has been associated with meningioma, and is thought to contribute to tumor development by disrupting these normal cellular processes. *TIMP3* is a gene that encodes a protein called tissue inhibitor of metalloproteinase 3, which inhibits the activity of enzymes that break down extracellular matrix components. Methylation of the *TIMP* gene has been found to be a common event in meningioma, and it is thought to promote tumor growth and invasion by increasing the activity of these enzymes. DNA methylation of *CDKN2A*, *NDRG2*, and *TIMP3* genes can contribute to the development of meningioma (Shen et al., 2020).

### Epigenetic biomarkers of promoter DNA methylation in meningioma

Promoter methylation is a specific type of gene methylation. The promoter is a region of the gene where RNA polymerase and other transcription factors bind to initiate transcription. The promoter region tends to be rich in CpG sites, which can be targeted for methylation. Promoter DNA methylation biomarkers have been investigated in meningiomas as a potential tool for improving their classification and predicting their clinical behavior. Several studies have identified specific genes that are frequently promoter methylated in meningiomas. Promoter DNA methylation of these genes has been associated with a higher grade and recurrence of meningiomas, as well as poorer patient outcomes. The identification of these biomarkers in meningiomas has also the potential to improve their classification and provide valuable information for treatment decisions and may also provide new targets for the development of novel therapies for meningiomas.

### Single-gene promoter DNA methylation in meningioma

*TERT* (Telomerase Reverse Transcriptase) gene encodes the telomerase reverse transcriptase, which is involved in maintaining telomere length and cell immortalization. Methylation of the *TERT* promoter has been shown to be a prognostic biomarker for meningioma, with higher levels of methylation associated with a worse prognosis (Mirian et al., 2022). Promoter methylation of *TERT* occurs during malignant transformation of meningiomas. *TERT* promoter methylation, intermediate, and malign MC were associated with reduced progression-free survival (PFS) and overall survival (OS). *TERT* is an independent significant prognostic factor for PFS. *TERT* promoter mutations have been found to linked with poor prognosis and cell immortalization in meningioma (Table 2; Goutagny et al., 2014). *TERT* promoter mutations were associated with a marked increase in *TERT* expression. Thus, *TERT* promoter mutations are essential genetic modifications complicated in malignant progression of meningiomas and can be used as a biomarker to identify meningiomas during malignant transformation (Goutagny et al., 2014). The analysis of mutant and DNA methylation of *TERT* promoter may facilitate personalized therapy in meningioma patients (Majchrzak-Celińska et al., 2013). Moreover, *hTERT* expression is found in meningiomas but independent of promoter methylation. In meningeal tumors, *hTERT* promoter methylation is more common than mutations and in meningiomas (Table 2; Fürtjes et al., 2016). *TERT* and *hTERT* promoter methylation plays an important role during oncogenesis of meningiomas with implications for diagnosis and precise treatment (Stögbauer et al., 2020).

**Table 2:** Promoter methylation and meningioma risk.

Target gene	WHO classification	Methylation classification	Function	References
Single gene DNA methylation				
*TERT*	Grade I, II and III	Int-A, Int-B, Mal	*TERT* promoter mutation is linked with higher grades of meningiomas.	Goutagny et al. (2014) and Gendreau et al. (2017)
*hTERT*	Grade I, II and III	All methylation	The promoter methylation of *HTERT* was positively correlated with WHO grade and *hTERT* expression.	Stögbauer et al. (2020)
*MGMT*	Grade I, II and III	Ben-2, Ben-3	More aggressive clinical behavior and higher risk of recurrence but also no convincing clinical results to test such tumors.	Das et al. (2020) and Korshunov et al. (2017)
*uPA*	Grade I, II and lll	Ben-2, Ben-3	*uPA* expression in meningiomas might be partially controlled by promoter methylation.	Kandenwein et al. (2011)
*MLH1*	Grade I, II and III	All methylation	Hypermethylation of the promoter of MLH1 is associated with the tumor grade	Chen et al. (2012)
*DLC1*		All methylation	*DLC1* encodes GTPase-activating protein with a tumor suppressor activity.	Bujko et al. (2016)
*TP73*	Grade I, II and III		TP73 promoter methylation as a biomarker for meningiomas	Suppiah et al. (2019)
*TIMP3*	Grade I, II and III	All methylation	Higher-grade meningiomas	Suppiah et al. (2019)
Multiple gene promoter DNA methylation				
*NF2, ARID1A*	Grade III (rhabdoid meningioma)	All methylation	Meningioma development and progression	Bujko et al. (2017)
*MGMT, CDKN2A, GSTP1*, and *THBS1*	Grade I, II and III	All methylation	*MGMT* (DNA repair), *CDKN2A* (cell cycle control), *GSTP1* (detoxification), and *THBS1* (angiogenesis inhibitor).	Aydemir et al. (2012)
*MGMT, RASSF1A, p15INK4B, and p14ARF*		All methylation	Moreover, the gene promoter methylation profiles observed in serum, in most cases, matched the methylation profiles detected in paired tumor samples.	Majchrzak-Celińska et al. (2013)
*MGMT, CDKN2A, GSTP1, and THBS1*	Grade I, II and III	All methylation	Meaningioma grade, recurrence, and response to chemotherapy	Aydemir et al. (2012)
*TRAF7, KLF4, NF2, TRAKL, ARID, AKT1*	Grade I, II	Ben-2, Int-B, Mal	TRAF7, KLF4, and TRAKL mutation genotype were associated with improved PFS and OS	Berghoff et al. (2022)

Progression-free survival (PFS) and overall survival (OS); AKT1, v-akt murine thymoma viral oncogene homolog 1; ARID, AT-rich interactive domain-containing protein; CDKN2A, Cyclin-dependent kinase inhibitor; DLC1, deleted in liver cancer 1; GSTP1, glutathione S-transferase pi 1; hTERT, human telomerase reverse transcriptase; KLF4, Krüppel-like factor; MGMT, o-6-methylguanine-DNA methyltransferase; MLH1, MutL homolog 1; NF2, neurofibromin 2; p14ARF, p14 alternate reading frame; p15INK4B, cyclin-dependent kinase inhibitor 2B; RASSF1A, Ras association domain-containing protein; TERT, tomerase reverse transcriptase; THBS1, thrombospondin 1; TIMP3, tissue inhibitor of metalloproteinases 3; TP73, tumor protein p73; TRAF7, TNF receptor-associated factor 7; TRAKL, trafficking kinesin-binding protein 1-like; uPA, urokinase-type.

O6-methylguanine-DNA methyltransferase (*MGMT*) promoter methylation was found in grade I, II, and III tumors. Methylation of *MGMT* gene promoter is a confirmed predictor in patients with glioblastoma. However, the methylation of the *MGMT* promoter occurs at a low rate in meningiomas. There was an increase, in *MGMT* methylation with a rise in the tumor grade. Higher methylation levels can be found in the male gender (Das et al., 2020). The clinical test for *MGMT* promoter methylation is still lacking (Panagopoulos et al., 2018). The methylation of the MGMT promoter is uncommon, or occurs at a low frequency in meningiomas. The study used pyrosequencing analysis to determine the MGMT promoter methylation status in 61 meningiomas. Only two tumors (3%) had a mean methylation frequency higher than the cut-off value of 10% for the four CpG sites examined. There is no convincing rationale to test such tumors for their MGMT methylation status in a clinical setting. However, the three most commonly used methods for detection of MGMT methylation are methylation-specific polymerase chain reaction (MSP), qPCR or the similar MethyLight methylation-specific quantitative real-time qPCR (MethyLight qMSP), and pyrosequencing. Each of these methods has its own limitations and may produce false-positive as well as false-negative results under some circumstances. Therefore, the clinical test for MGMT promoter methylation is still lacking in terms of its reliability and applicability in a clinical setting, particularly for meningiomas. There is a need for more accurate and reliable testing methods for MGMT promoter methylation in meningiomas. Enhanced promoter methylation of the *uPA* promoter is observed to link significantly with lower levels of *uPA*. *uPA* expression in meningiomas might, in part, be controlled by promoter methylation (Table 2; Kandenwein et al., 2011). The mismatch repair genes human mutL homolog 1 (hMLH1) has no enzymatic activity and exerts its function as a “molecular matchmaker,” which recruits other DNA repair proteins to the mismatch repair complex. Meningiomas shows hypermethylation of the hMLH1 promoter (Table 2; Chen et al., 2012).

DNA hypermethylation was associated with lower levels of deleted in liver cancer 1 (D*LC1*) expression in meningiomas. The *DLC1* isoform 1 is the moste expressed in normal tissues and considerably reduced in meningioma tissue, which is caused by hypermethylation of CpG dinucleotides within the isoform promoter region (Bujko et al., 2016).

*TIMP3* is commonly hypermethylated in higher grade of meningiomas, which leads to its downregulated transcription levels and prevents its anti-tumor activities. About 70–80% of high-grade meningiomas have *TP73* promoter methylation but not in grade I meningioma, suggesting *TP73* promoter methylation is a marker for higher-grade meningiomas (Table 2; Suppiah et al., 2019).

### Multiple-gene promoter DNA methylation in meningioma

Increased methylation of the promoter region of the *NF2* gene was associated with a higher risk of meningioma recurrence. DNA methylation of the *ARID1A* promoter region is frequently increased in meningiomas, which can lead to decreased expression of the gene. DNA methylation of the *ARID1A* promoter was significantly higher in meningioma compared to normal brain tissue, which is associated with larger tumor size and higher grade (Bujko et al., 2017). The genes *MGMT*, *CDKN2A*, glutathione S-transferase pi 1 (*GSTP1*), and thrombospondin 1 (*THBS1*) have all been implicated in the development and progression of meningiomas, and their promoter regions have been studied for methylation profiles. In meningiomas, *CDKN2A* promoter methylation has been observed in a subset of tumors, particularly those with aggressive features. *GSTP1* is a gene that encodes an enzyme involved in detoxification processes. *GSTP1* promoter region has been associated with reduced expression of the protein and increased susceptibility to meningioma, and whose promoter methylation has been observed in those with a higher risk of recurrence. *THBS1* encodes for a glycoprotein associated with cell adhesion, migration, and angiogenesis. Methylation in promoter regions of *MGMT*, *CDKN2A*, and *GSTP1* but not *THBS1* genes are found in meningioma. Based on their WHO grade, more higher grade has hypermethylation in the promoter regions of these genes (Table 2; Aydemir et al., 2012). These genes have all been implicated in the development and progression of meningiomas, and their promoter methylation profiles have been associated with meningioma grade, recurrence, and response to chemotherapy.

Compared to metastatic brain tumor patients, the patients with glial tumors have a higher frequency of DNA hypermethylation. Among, the methylation profiles of *MGMT*, Ras association domain-containing protein (*RASSF1A*), *p15INK4B*, and *p14ARF* genes, the hypermethylation of *RASSF1A* differentiated primary from metastatic brain cancer (Table 2; Majchrzak-Celińska et al., 2013). TNF receptor-associated factor 7 (*TRAF7*), trafficking kinesin-binding protein 1-like (*TRAKL*), *KLF4*, *NF2*, *ARID1A*, *AKT1* are genes that have been reported to be frequently methylated in meningioma. *TRAF7* and *KLF4* promoter methylation has been found to be associated with meningioma recurrence and higher tumor grade. *NF2* promoter methylation has been found to be associated with reduced expression of the NF2 protein and with meningioma progression. Promoter DNA methylation of *TRAF7, KLF4, NF2, TRAKL, ARID1A, and AKT1* and meningioma suggests that aberrant DNA methylation of these genes may be involved in the development and progression of meningioma (Berghoff et al., 2022).

### Potential clinical applications of DNA methylation inhibitors

Findings from DNA methylation of meningioma biomarkers have dramatically shifted our understanding of meningiomas. Nevertheless, the clinical application of these biomarkers is still extremely limited. Subclassification of meningiomas by methylation methods and prediction of prognosis remains not beyond a research stereotype. The epigenetics of meningioma has led to a number of current clinical trials aiming to improve meningioma therapy by targeting the methylated biomarkers. A clinical trial has been conducted to investigate the effectiveness of four different drugs in treating patients with progressive meningiomas, including Vismodegib, FAK inhibitor GSK2256098, Capivasertib, and Abemaciclib (ClinicalTrials.gov Identifier: NCT02523014). Vismodegib, an inhibitor of SMO, has been performed in trial for SMO-mutated meningiomas. Capivasertib is an investigational drug that targets AKT, which is involved in the regulation of cell growth and survival, and shown antitumor activity in preclinical studies. Abemaciclib is a drug that inhibits cyclin-dependent kinases 4 and 6 (CDK4/6), which are involved in the regulation of cell cycle progression. It has been approved by the FDA for the treatment of breast cancer and is being studied for the treatment of other cancers. However, none of them is DNA methylation inhibitor. DNA methylation inhibitors are a class of drugs that target the DNA methylation process and have shown promise in the treatment of several subtypes of meningiomas.

### Nucleoside analogs

Nucleoside analogs, such as Azacitidine, Decitabine and FdCyd, are DNA methylation inhibitors that are used in the treatment of various types of cancers (Sun et al., 2022). Azacitidine is a DNA methylation inhibitor that is commonly used in the treatment of myelodysplastic syndromes and acute myeloid leukemia. Azacitidine inhibited the meningioma cells and induced cell cycle arrest and apoptosis (programmed cell death). The study suggests that Azacitidine may have potential as a therapeutic agent for the treatment of meningiomas (Table 3; Das et al., 2020). Decitabine is a drug that inhibits DNA methylation, a process that is involved in the regulation of gene expression. There is limited research on the use of decitabine specifically for meningioma. Decitabine may have potential as a treatment for meningioma. Decitabine reduced the growth of meningioma cells *in vitro*, and it may be effective in treating recurrent or progressive meningiomas (Stögbauer et al., 2020; Goyal-Honavar et al., 2022). Decitabine decreases proliferation and viability in high-grade meningioma but not in benign meningioma (Stögbauer et al., 2020).

**Table 3:** DNA methylation inhibitors.

Therapeutic agent	Drug name	Functional mechanism	Research levels	References
Nucleoside	Azacitidine	It is involved in the synthesis of RNA or DNA at high concentrations; Inhibition of DNMT	Meningioma cells	Das et al. (2020)
Nucleoside	Decitabine	At high concentrations it can lead to blocked DNA synthesis and cytotoxicity; at low concentrations it leads to changes in gene expression profiles.	Meningioma cells	Goyal-Honavar et al. (2022) and Stögbauer et al. (2020)
Nucleoside	FdCyd	FdCyd inhibits DNA methylation by the same mechanism as decitabine.	NA	Hu et al. (2021)
Nucleoside	Zebularine	A nucleoside analog forms a covalent complex with DNMT to inhibit DNA methylation	Meningioma cells	Velpula et al. (2013)
Nucleoside	RX-3117	RX-3117 can be further phosphorylated to its diphosphate form, known as RX-DP. RX-DP has been shown to inhibit the activity of thymidylate synthase, which is an enzyme required for the synthesis of thymidine, a building block of DNA. By inhibiting thymidylate synthase, RX-DP can lead to decreased levels of thymidine, which can affect DNA replication and cell division, leading to cell death.	NA	Udvaros et al. (2015)
Nucleoside	Cladribine	They can reduce the activity of S-adenosyl-L-homocysteine hydrolase, which regulates methylation reactions.	NA	Molica et al. (2020)
Nucleoside	Fludarabine			
Non-nucleoside	Procainamideand procaine	It binds tightly to CpG island dense regions of DNA thereby interfering with the binding of DNMT to DNA	Clinical	Bird (1929)
Non-nucleoside	RG108	Non-covalent binding to the DNMT active site to achieve a block to DNA methylation.	NA	Bujko et al. (2017)
Non-nucleoside	EGCG	It binds non-covalently to the catalytic active site of DNMT to inhibit the methylation catalytic activity of DNMT.	NA	Bujko et al. (2017)
Non-nucleoside	MG-98	Acts on the mRNA of DNMTI and radically inhibits the expression and synthesis of DNMTI.	NA	Bujko et al. (2017)
Non-nucleoside	Guadecitabine	The constitutive methylation level of the CTA promoter in cancer cells was significantly reduced.	NA	Dan et al. (2019)
Non-nucleoside	SGI1027	Induction of degraded DNMTs for demethylation.	NA	Yuzawa et al. (2016)
Non-nucleoside	Nanaomycin A	It reduces global methylation levels.	NA	Smith (2015)
Non-nucleoside	Mithramycin A	The antibiotic mithramycin A (MMA) is a potential DNMT1 inhibitor, which could reduce the CpG island methylation	NA	Fukami et al. (2016)
Non-nucleoside	Hydralazine	It reduces global methylation levels.	NA	Gendreau et al. (2017)
Non-nucleoside	Genistein	It reduces global methylation levels.	NA	Clark et al. (2016)
Non-nucleoside	Equol	It decreases the methylation of the cytosine phosphate guanine (CpG) islands.	NA	Fatima et al. (2020)
Non-nucleoside	Curcumin	Induces hypomethylation and cellular apoptosis.	Meningioma cells	Curic et al. (2013) and Yamazaki et al. (2021)
Non-nucleoside	Disulfiram	As a DNMT1 inhibitor, it can reduce global 5meC proportion.	NA	Abedalthagafi et al. (2016)
Non-nucleoside	Resveratrol	It regulates ERα promoter DNA methylation.	Meningioma cells	Farhan et al. (2019) and Hu et al. (2019)
Non-nucleoside	Caffeic acid	It regulates global DNA methylation.	NA	Hernandes et al. (2020)
Non-nucleoside	Chlorogenic acid	It regulates global DNA methylation.	NA	Hernandes et al. (2020)
Non-nucleoside	Dabrafenib	BRAF inhibitor	Clinical	Wen et al. (2019)
Non-nucleoside	AZD5363	AKT inhibitor AZD5363	NA	John et al. (2022)

Decitabine, 5-aza-2′-deoxycytidine; EGCG, epigallocatechin-3-gallate; FdCyd, 5-fluoro-2′-deoxycytidine.

FdCyd (5-Fluoro-2′-deoxycytidine) is a nucleoside analog that has been used as a chemotherapy drug to treat different types of cancers. FdCyd is well-known DNA methylation inhibitors that has been extensively used in research and clinical settings. The compound is incorporated into DNA during replication and interfere with DNA methyltransferases (Hu et al., 2021). However, the preclinical studies about the effects of FdCyd on meningiomas in humans have not been studied yet. Zebularine is a small molecule compound that functions as a DNA methylation inhibitor. Zebularine works by inhibiting the activity of DNA methyltransferase enzymes. Zebularine has been studied for its potential use in cancer therapy, as it can help to reactivate tumor suppressor genes that have been silenced through DNA methylation. Recent research has investigated the potential use of zebularine as a therapeutic agent for meningiomas. Zebularine was found to inhibit the growth of meningioma cells and induce cell death *in vitro*. The researchers also found that zebularine treatment led to changes in the expression of genes involved in cell proliferation and apoptosis. The drug was able to inhibit cell proliferation and induce cell death in a dose-dependent manner (Velpula et al., 2013).

As adenosine analogs DNMTi, cladribine and fludarabine can inhibit DNA methylation from three major aspects. They can incorporate into DNA sequences to form triphosphate deoxynucleotide to inhibit DNA synthesis (Greene et al., 2020; Ball et al., 2022). They can control proliferating cell nuclear antigen (PCNA) to induce DNMT1 activity on semi-methylated DNA by increasing p21 level (Zhang et al., 2022). They can reduce the activity of S-adenosyl-L-homocysteine (SAH) hydrolase, which regulates methylation reactions, resulting in the decrease in the levels of both DNA and histone methylation (Molica et al., 2020). Cladribine is a nucleoside analog of deoxyadenosine. A chlorine substitution in the purine ring protects cladribine against degradation by adenosine deaminase. Cladribine is currently used for the treatment of multiple sclerosis (Rammohan et al., 2020). Cladribine was rapidly absorbed. Cladribine has good oral bioavailability, and has the potential to penetrate the blood–brain barrier (Ford et al., 2022). Fludarabine is a fluorinated derivative of adenosine, which can prevent the deamination of adenosine deaminase (Greene et al., 2020; Ball et al., 2022). Guadecitabine is a drug that belongs to the class of hypomethylating agents and is used for the treatment of various types of cancer, including myelodysplastic syndromes and acute myeloid leukemia. It works by incorporating into DNA and inhibiting the activity of DNA methyltransferase (Daifuku, 2019).

### Natural flavonoids and phenolic acid

Genistein is a natural isoflavone found in soybeans and has been shown to have various health benefits, including reducing the risk of meningioma by inhibiting DNA methylation. Genistein inhibited the growth of meningioma cell line *in vitro* and induced cell apoptosis (Clark et al., 2016). RX-3117 is a DNA methylation inhibitor that has shown promising results in a potential treatment for brain tumors (Udvaros et al., 2015). There is also limited research on the use of RX-3117 specifically for meningiomas. However, studies have suggested that DNA methylation inhibitors, in general, may be effective against meningioma. S-equol is a compound that is produced in the gut by the microbial metabolism of the soy isoflavone daidzein. S-equol can act as a DNA methylation inhibitor, and was found to decrease DNA methylation levels in breast cancer cells, leading to reactivation of tumor suppressor genes and reduced cancer cell growth. S-equol may inhibit DNA methyltransferases by interfering with their binding to DNA, while others have proposed that S-equol may affect the availability of the methyl donor molecule S-adenosylmethionine (SAM), which is required for DNA methylation (Hod et al., 2021).

Curcumin is a natural polyphenol compound found in the spice turmeric, and has been investigated for its potential antitumor properties. Curcumin can cause hypomethylation of the promoter region of the *BRCA1* gene by downregulating ten-eleven translocation 1 (TET1) gene, whereas curcumin-induced hypermethylation of SNCG by upregulating DNA methyltransferase 3 (DNMT3) and downregulating TET1. Curcumin has an dual function on DNA promoter methylation (Al-Yousef et al., 2020). However, the effects of curcumin on meningioma have not been reported yet. Disulfiram is a drug that has been investigated for its potential anticancer properties. Disulfiram can act as a DNMT1 inhibitor, leading to global DNA hypomethylation and reactivation of silenced tumor suppressor genes. Disulfiram treatment was found to decrease DNMT1 expression and activity in cancer cells, resulting in reactivation of silenced tumor suppressor genes and reduced cancer cell growth (Zhang et al., 2022). Curcumin has strong antitumor functions in meningioma cells and may be a potential drug for the pharmacological treatment of meningiomas (Curic et al., 2013). FdCyd inhibits DNA methylation by the same mechanism as decitabine. It can inhibit DNA methylation by integrating into DNA sequences in the form of triphosphate or binding to DNMT covalently. FdCyd is currently in phase II clinical trials for the treatment of solid tumors (Hu et al., 2021).

Resveratrol is a naturally occurring polyphenol found in various plants, including grapes and berries, and has been investigated for its potential as a DNA methylation inhibitor in the treatment of cancer, including meningioma. Studies have shown that resveratrol can inhibit the activity of DNA methyltransferases and lead to global DNA hypomethylation in cancer cells, including meningioma cells. In addition, resveratrol has been shown to induce the reactivation of silenced tumor suppressor genes, such as RARβ, in cancer cells. Resveratrol has shown promise as a potential therapeutic agent for various types of cancer (Farhan et al., 2019). Resveratrol inhibits the proliferation and induces apoptosis in meningioma cells by upregulating miR-34a-3p (Hu et al., 2019). Caffeic acid is a naturally occurring phenolic acid found in various plants, including coffee and fruits, and has been investigated for its potential as a DNA methylation inhibitor in the treatment of cancer, including meningioma. Caffeic acid can inhibit the activity of DNA methyltransferases and lead to global DNA hypomethylation in meningioma cells. In addition, caffeic acid has been shown to induce the reactivation of silenced tumor suppressor genes, such as p16 and RASSF1A, in cancer cells (Hernandes et al., 2020).

Chlorogenic acid is a natural phenolic acid found in various plants, including coffee, fruits, and vegetables, and has been investigated for its potential as a DNA methylation inhibitor in the treatment of meningioma. Chlorogenic acid can inhibit the activity of DNA methyltransferases and lead to global DNA hypomethylation in meningioma cells (Hernandes et al., 2020).

### Antibiotics

Nanaomycin A is a small molecule that has been investigated for its potential as a DNA methylation inhibitor in the treatment of cancer. It works by inhibiting the activity of DNA methyltransferases, which are enzymes that add methyl groups to DNA, leading to changes in gene expression. To date, there is no specific research on the potential effects of nanaomycin A on meningiomas. Nanaomycin A treatment inhibited the growth and proliferation of glioblastoma cells *in vitro*. The authors of the study suggested that the anti-tumor effects of nanaomycin A may be due to its ability to induce hypomethylation and reactivation of silenced tumor suppressor genes in glioblastoma cells (Smith, 2015). Mithramycin A (MMA) is a small molecule antibiotic that has been investigated for its potential as a DNA methylation inhibitor in the treatment of cancer. It works by inhibiting the activity of DNA methyltransferases, which are enzymes that add methyl groups to DNA, leading to changes in gene expression. There is limited research on the potential effects of MMA on meningiomas. MMA treatment was found to induce hypomethylation and reactivation of silenced tumor suppressor genes, such as *RASSF1A*, in meningioma cells. The anti-tumor effects of MMA in meningiomas may be due to its ability to induce hypomethylation and reactivation of silenced tumor suppressor genes in meningioma cells (Fukami et al., 2016).

### Others

SGI-1027 is a small molecule that has been investigated for its potential as a DNMT inhibitor, which induces the degradation of DNMT1 and reactivate tumor suppressor genes (Champeaux et al., 2019). MMA (3-deazaneplanocin A, DZNep) is a small molecule inhibitor of the enzyme S-adenosyl-L-homocysteine hydrolase (SAHH) that has been investigated for its potential as a DNMT1 inhibitor. SAHH is an enzyme that regulates cellular levels of S-adenosylhomocysteine (SAH), which is a byproduct of DNA methylation and is known to inhibit the activity of DNA methyltransferases. By inhibiting SAHH, MMA can increase cellular levels of SAH, which in turn inhibits the activity of DNMT1 and can lead to global DNA hypomethylation. MMA has been shown to be particularly effective at reducing the CpG island methylation of tumor suppressor genes, such as SLIT2 and TIMP-3, which are often silenced in cancer cells. In preclinical studies, MMA has shown promise as a potential therapeutic agent for various types of cancer (Cao et al., 2023).

RG108 is a small molecule inhibitor of DNA methyltransferases that has been investigated for its potential as a DNA methylation inhibitor in the treatment of cancer. It has been shown to reduce DNA methylation levels, leading to the reactivation of silenced tumor suppressor genes, and has shown promise as a potential therapeutic agent for various types of cancer (Bujko et al., 2017).

Dabrafenib is a small molecule inhibitor of BRAF, a protein that is commonly mutated in various types of cancer, including melanoma and some types of brain tumors. While dabrafenib is not a direct DNA methylation inhibitor, it has been investigated for its potential indirect effects on DNA methylation. Studies have shown that dabrafenib treatment can induce hypomethylation of the SOX10 gene, which is a transcription factor that is involved in the regulation of various cellular processes, including cell differentiation and proliferation. SOX10 is a target gene of BRAF, and its expression is commonly dysregulated in BRAF-mutant cancers (Wen et al., 2019). AZD5363 is a small molecule inhibitor of AKT, a protein that is commonly overexpressed or mutated in meningiomas (John et al., 2022). While AZD5363 is not a direct DNA methylation inhibitor, it has been investigated for its potential indirect effects on DNA methylation. AKT can regulate the activity of DNA methyltransferases and lead to changes in DNA methylation patterns in cancer cells. In addition, AKT activation has been associated with hypermethylation of certain genes, including tumor suppressor genes in meningioma.

## Limitations and future development

Exploring DNA methylation biomarkers in meningiomas is an important area of research that can provide valuable information about the diagnosis, prognosis, and response to treatment of meningioma. It may lead to the development of new targeted therapies that can improve outcomes for meningioma patients.

DNA methylation of ARID1A may be a useful biomarker for meningiomas and could potentially be targeted for therapy in future. Additionally, a better understanding of the epigenetic regulation of ARID1A in meningiomas may help identify new therapeutic targets and improve patient outcomes, especially the DNA methylation patterns of combined meningioma biomarkers can provide more clear grades, tissue subtypes and exact therapy targets. While these findings are promising, more research is needed to fully understand the potential of the epigenetics for treating meningioma. It is important to note that the most DNA methylation inhibitors (FdCyd, RX-3117, Cladribine, RG108, EGCG, MG-98, Guadecitabine, SGI1027, NMA, MMA, Hydralazine, Genistein, Equol, Disulfiram, Caffeic acid, Chlorogenic acid and AZD5363, Table 3) can still be unavailable or unproved for meningioma and have side effects, such as low blood cell counts and increased risk of infection (Abedalthagafi et al., 2016; Wei et al., 2021; Liu et al., 2023), so it should be carefully approved before clinical application.

Mutations or alterations in several gene DNA methylation have been associated with the development of meningiomas, including NF2, TRAF7, KLF4, AKT1, p73, MGMT, RASSF1A, hoxa, SMO, PI3KCA, POLR2A, SUFU, SMARCB1, SMARCE1, CDKN2A, and CDKN2B so on. The studies have suggested that DNA methylation patterns in meningiomas, although the specific relationship between these alterations and the development or progression of meningiomas is not yet fully understood. Future research is needed to fully understand the relationship between DNA methylation of these genes and meningioma development and progression, these findings suggest that DNA methylation patterns may be a potential biomarker for meningioma diagnosis, prognosis, and treatment response. These findings could potentially be integrated into clinical practice in the future, subject to additional studies and clinical trials. Additionally, a clinical trial is currently underway to investigate the use of these inhibitors in combination with radiation or other therapies for meningioma treatment.

Genomic and epigenomic studies, including those focused on DNA methylation, have provided new insights into the molecular basis of these tumors. With the advent of Next-Generation Sequencing (NGS), scientists can now profile DNA methylation across the entire genome, allowing a more comprehensive understanding of its role in meningioma development and progression. Capper et al., presented a DNA methylation-based approach for classification of central nervous system tumors, including meningiomas. This work provided strong evidence that molecular classification based on DNA methylation profiles can outperform traditional histopathological methods (Capper et al., 2018). DNA methylation-based classification of central nervous system tumors. Nature, 555: 469–474. Nassiri et al. developed and validated a DNA methylation-based predictor of recurrence risk in meningioma. It found that the predictor was independently associated with RFS after controlling for tumor grade, extent of resection, and burden of copy number alterations. The predictor was able to stratify patients with WHO grade I, II, and III tumors into higher and lower risk groups, with significant differences in median RFS between the groups (Nassiri et al., 2019). DNA methylation profiling to predict recurrence risk in meningioma: development and validation of a nomogram to optimize clinical management. Neuro-oncology, 21: 901–910. Choudhury et al. used a DNA methylation analysis method called SeSAMe to identify three groups of meningiomas. It found that these groups could be independently validated and were associated with distinct clinical outcomes. The study also compared SeSAMe with another analysis method that does not control for the influence of copy number variations on beta values and found that SeSAMe provided more accurate grouping and better prediction of clinical outcomes (Choudhury et al., 2022). Meningioma DNA methylation groups identify biological drivers and therapeutic vulnerabilities. Nature genetics, 54: 649–659. This work revealed that some methylation changes in meningiomas are associated with patient age and tumor location, demonstrating the complex interplay between genetics, epigenetics, and environmental factors. These studies, among others, illustrate the power of NGS to yield new insights into the molecular basis of meningiomas. However, much work remains to be done before this knowledge can be fully integrated into clinical practice, and ongoing research is aimed at deepening our understanding of these complex epigenetic changes. It is hoped that these advances will lead to better diagnostic tools, prognostic markers, and potentially new treatment strategies for meningiomas.

## Conclusion

DNA methylation is an epigenetic modification that plays a crucial role in gene expression regulation. Methylation patterns in DNA have been shown to be associated with various subtypes of meningioma and DNA methylation markers have been identified as potential diagnostic and prognostic biomarkers. Here are some attributes and limitations of DNA methylation meningioma biomarkers.

### Attributes

High sensitivity and specificity: DNA methylation biomarkers have been shown to have high sensitivity and specificity for the diagnosis of meningioma. In terms of DNA methylation and its sensitivity and specificity in meningiomas, one study reported that DNA methylation profiling of meningiomas had a sensitivity of 92.1% and a specificity of 97.2% (Sahm et al., 2017). However, more research might be needed to fully understand the role and impact of DNA methylation in meningiomas. Moreover, DNA methylation profiling has shown to be helpful in predicting the course of the disease and the recurrence risk, which might play a critical role in deciding the appropriate treatment strategy. A molecularly integrated classification based on DNA methylation profiling has high prognostic relevance. The results of the study showed that the methylome-based predictor of 5-year recurrence-free survival (RFS) performed favorably compared with a grade-based predictor. The predictor was able to distinguish risk groups (lower and higher risk) in all three validation cohorts. Moreover, the models developed and validated in this study provide important prognostic information not captured by previously established clinical and molecular factors. This could be used to individualize decisions regarding postoperative therapeutic interventions, in particular whether to treat patients with adjuvant radiotherapy versus observation alone (Nassiri et al., 2019). They can differentiate meningioma from normal brain tissue and other brain tumors with high accuracy. Non-invasive: DNA methylation biomarkers can be detected in body fluids such as blood and cerebrospinal fluid, making them a non-invasive diagnostic tool. Early detection: Methylation changes can occur early in the development of meningioma, making them potential early detection biomarkers. Tumor subtype classification: DNA methylation markers can distinguish between different meningioma subtypes, which have different clinical outcomes and treatment options.

WHO classification grading and DNA methylation: Emerging evidence suggests that DNA methylation status is a significant factor in meningioma grading. One study retrospectively collected 497 meningioma samples and found that DNA methylation profiling could distinguish six distinct clinically relevant methylation classes. Notably, compared with WHO grading, classification by individual and combined methylation classes more accurately identified patients at lower risk of recurrence among WHO grade II meaningioma (Sahm et al., 2017). DNA methylation profiling has shown high prognostic relevance, demonstrating an ability to further stratify WHO grade II meningiomas into distinct prognostic subgroups. This adds a layer of complexity to the WHO grading but also offers the potential for more personalized prognosis and treatment strategies. The new WHO classification takes into account this burgeoning understanding of methylation status, providing a more nuanced approach to meningioma classification. However, there is a need for further study to completely integrate these findings into the classification and management of meningiomas.

### Limitations

Tissue specificity: DNA methylation markers can show tissue-specific patterns, which may limit their use as biomarkers in other tissues or organs. Variability: DNA methylation patterns can vary between individuals, and this variability may limit the accuracy of the biomarkers for meningioma diagnosis and prognosis. Limited knowledge: The knowledge of DNA methylation biomarkers for meningioma is limited, and more research is needed to identify new biomarkers with higher sensitivity and specificity. Clinical implementation: The use of DNA methylation biomarkers in clinical practice requires standardized protocols for sample collection, processing, and analysis, which can be challenging to implement in some settings. The most DNA methylation inhibitors have not been approved in meningioma yet.

In summary, DNA methylation markers have shown promise as diagnostic and prognostic biomarkers for meningioma, but their clinical utility is limited by tissue-specificity, variability, limited knowledge, and the need for standardized protocols. Further research is needed to improve the accuracy and clinical implementation of DNA methylation biomarkers for meningioma.

## Author contributions

ZL, YG, and JZ: conceptualization. JZ, LH, and HZ: methodology and writing—original draft preparation. ZL, JZ, and LH: resources. LH, YG, and HZ: writing—review and editing. HZ: supervision. All authors have read and agreed to the published version of the manuscript and approved the final version of the manuscript.

## Conflict of interest

The authors declare that the research was conducted in the absence of any commercial or financial relationships that could be construed as a potential conflict of interest.

## Publisher’s note

All claims expressed in this article are solely those of the authors and do not necessarily represent those of their affiliated organizations, or those of the publisher, the editors and the reviewers. Any product that may be evaluated in this article, or claim that may be made by its manufacturer, is not guaranteed or endorsed by the publisher.
